# [Retracted] miR-331-3p suppresses cell invasion and migration in colorectal carcinoma by directly targeting NRP2

**DOI:** 10.3892/ol.2026.15738

**Published:** 2026-07-01

**Authors:** Hongye Zhang, Ruiyu Wang, Mingxia Wang

Oncol Lett 18: 6501–6508, 2019; DOI: 10.3892/ol.2019.11029

Following the publication of the above paper, it was drawn to the Editor's attention by a concerned reader that, regarding the cell migration and invasion assay data shown in Fig. 2C on p. 6504, the Invasion, SW480/NC and Migration, SW480/NC panels showed an overlapping section of data, such that these data panels, which were intended to show the results of differently performed experiments had been derived from the same original source. In addition, the Invasion, SW480/NC data panel was also found to contain an overlapping section with the Invasion, SW480/mir-331-3p inhibitor+NRP2 siRNA data panel in Fig. 5C. A concern was also raised that the miRNA results were erroneously stated to have been normalized to U6 snRNA levels according to the qPCR analysis, whereas the sequences reported for the primers were not for U6, but for a messenger RNA, LSM5. Moreover, upon performing an independent analysis of the data in this paper in the Editorial Office, it also came to light that, regarding the western blot experiments featured in Figs. 3 and 4 on p. 6505, a unique GAPDH protein band featured in Fig. 3A was strikingly similar to a GAPDH protein band featured in Fig. 2C, whereas the other GAPDH protein band in Fig. 3A appeared to be strikingly similar to the NRP2 protein band located in the ‘NC’ lane of the gel in Fig. 4D.

Given the large number of potentially anomalous issues identified with the abovementioned figures in this paper, the Editor of *Oncology Letters* has decided that it should be retracted from the Journal on account of a lack of confidence in the presented data. The authors were asked for an explanation to account for these concerns, but the Editorial Office did not receive a reply. The Editor apologizes to the readership for any inconvenience caused.

